# Long-term follow-up after en bloc resection and reconstruction of a solitary paraganglioma metastasis in the first lumbar vertebral body: a case report

**DOI:** 10.1186/1752-1947-5-45

**Published:** 2011-02-01

**Authors:** Alexander Richter, Henry F Halm, Thomas Lerner, Ulf R Liljenqvist, Markus Quante

**Affiliations:** 1 Spine Center Hamburg, Asklepios Klinik St. Georg, Lohmühlenstrasse 5, 20099 Hamburg, Germany; 2 Department of Spine Surgery and Scoliosis Center, Klinikum Neustadt, 23730 Neustadt i. H., Germany; 3 Department of Spine Surgery, St. Franziskus Hospital, 48145 Münster, Germany

## Abstract

**Introduction:**

Paragangliomas are rare tumors that originate from the autonomic nervous system-associated paraganglia. They metastasize infrequently. Malignancy can only be demonstrated by the presence of chromaffin tissue at sites where it usually is not present, such as bone, lung or liver, or local recurrence after total resection of a primary mass. Paragangliomas within the central nervous system are usually intradural near the conus medullaris. The metastatic spread of a retroperitoneal paraganglioma to a vertebral body is extremely rare, and there are only a few cases reported in the literature.

**Case presentation:**

We report the case of a 16-year-old Caucasian girl who had undergone resection of a retroperitoneal paraganglioma that measured 15 × 11.5 × 9.5 cm. After further staging, a solitary metastatic paraganglioma was detected in the first lumbar vertebral body. After initial chemotherapy, marginal en bloc resection and reconstruction were performed followed by radiotherapy. Histologic examination of the specimen revealed that the tumor cells did not show any response to preoperative chemotherapy, which is in line with a few other reports in the literature. Ten years after operative treatment, the patient is free of complaints, very satisfied with the result and without signs of local recurrence or distant metastases.

**Conclusion:**

We recommend en bloc spondylectomy and local radiotherapy in the treatment of solitary spinal metastatic paragangliomas.

## Introduction

Paraganglioma is a rare tumor that originates from the autonomic nervous system-associated paraganglia. Approximately 90% of paragangliomas arise from the adrenal medulla, carotid body and glomus jugulare [1-3]. These metastasize infrequently. Within the central nervous system, the majority of paragangliomas arise intradurally in the area of the cauda equina [2]. For extra-adrenal retroperitoneal paragangliomas, a 50% rate of metastasis has been described [4,5]. Extra-adrenal paragangliomas are divided on the basis of their anatomic distribution into cervical, thoracic and intraabdominal tumors [6]. About 15% to 20% of childhood paragangliomas are extra-adrenal [7]. Metastatic spine involvement is uncommon, and if it occurs, it is generally intradural at the level of the cauda equina, very rarely within the vertebral bodies [1,3,8-17]. The individual behavior of paragangliomas is unpredictable because the fundamental characteristics of malignant neoplasms such as vascular invasion and extensive local invasion are of limited value in assessing neuroendocrine tumors [17].

We present one rare case of a solitary L1 metastatic paraganglioma, which was detected after removal of an intraabdominal paraganglioma. Preoperative chemotherapy, en bloc spondylectomy and postoperative radiotherapy were performed.

## Case presentation

An otherwise healthy 16-year-old Caucasian girl presented with a sudden onset of cramp like pain in the right lower abdomen. After examination, her gynecologist performed laparoscopy and found extreme varicosis of the internal genital tract but without further pathologic findings. To exclude thrombosis and consecutive collateral circulation, postoperative phlebography was done and showed excessive displacement of the inferior vena cava. A retroperitoneal tumor was suspected, and magnetic resonance imaging (MRI) revealed a tumor measuring 14 × 10 × 14 cm in the right abdomen with a craniodorsal shift of the kidney. Laboratory parameters, including tumor markers (24-hour urinary catecholamines and metabolites, dopamine, serum and plasma α-fetoprotein, neuron-specific enolase (NSE), β-human chorionic gonadotropin) were within normal ranges.

Explorative laparotomy was performed, and the retroperitoneal tumor was resected. The tumor weighed 817 g, and macroscopic examination demonstrated a thinly encapsulated neoplasm. The diagnosis of a paraganglioma was confirmed by histologic and immunohistologic examinations. Because vascular invasion and focal infiltration of the fibrous capsule could be shown, it was an R1 marginal resection.

**Figure 1:**
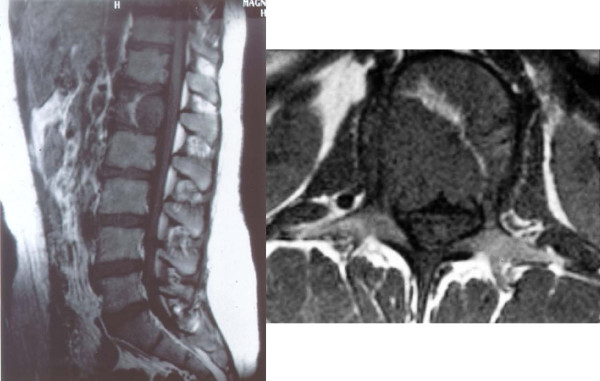
**Magnetic resonance image showing the metastatic lesion within the vertebral body with destruction of the posterior cortex, encroachment of the spinal canal and invasion of the left pedicle**.

The postoperative course was uneventful, but because of the potential malignant behavior of extra-adrenal paragangliomas, Tc-99-MDP (Tc-99m-methylene diphosphonate) and I-123-MIBG (123 I-metaiodobenzylguanidine) scintigraphy was performed 10 and 21 days postoperatively. An increased uptake in the first lumbar vertebra was noted and MRI showed a lesion in the left dorsal third of the L1 vertebral body (Figure 1). The supposed metastatic paraganglioma was confirmed by computed tomography- (CT-) guided needle biopsy. Chemotherapy was applied using a neuroblastoma protocol (NB 90 of the German Society of Paediatric Oncology and Haematology).

**Figure 2:**
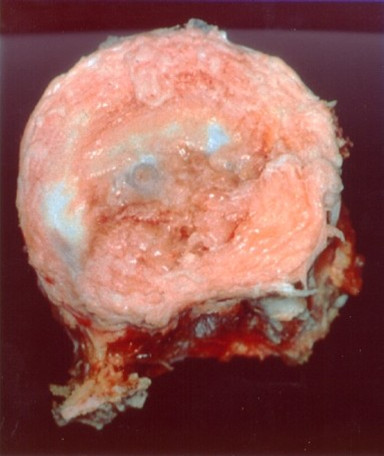
**En bloc resected vertebral body with the affected left pedicle left en bloc**.

**Figure 3:**
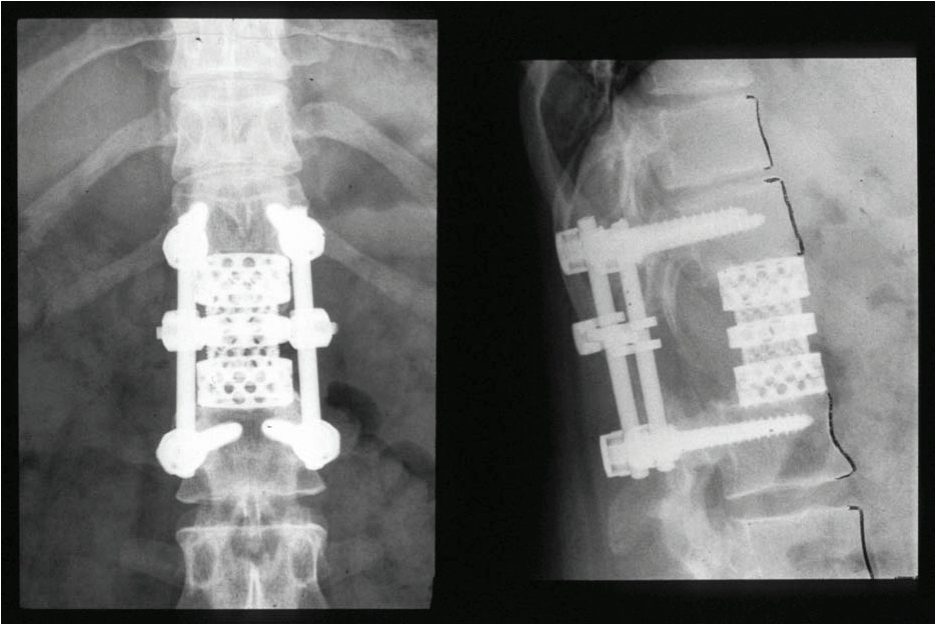
**Postoperative anteroposterior and lateral plane radiograph showing reconstruction with modular tumor cage and a pedicle-screw instrumentation**.

**Figure 4:**
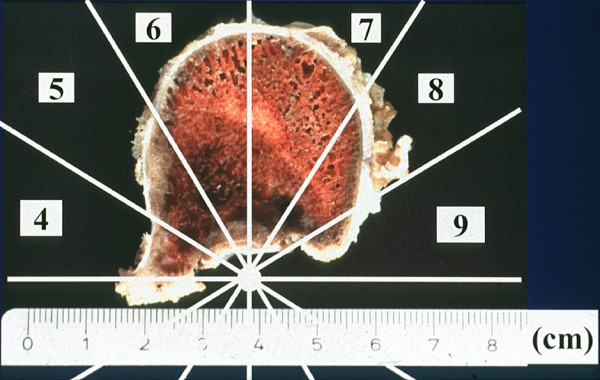
**Horizontal cut through the resected vertebral body**. Complete destruction of the posterior cortical lamellae with intact pseudocapsule. Metastatic lesion in zones 4 to 9 and layer B (intraosseous superficial), according to Boriani et al [23].

Five months later, combined posteroanterior en bloc resection of the L1 vertebra was performed. Because of partial infiltration of the left pedicle, it was left en bloc with the vertebral body (Figure 2). Reconstruction was performed with posterior transpedicular screw instrumentation and anterior reconstruction using a modular cage filled with autologous morselized rib grafts (Figure 3). Macroscopically, the cut surface of the vertebral body showed a reddish tumor in the left dorsolateral part of the vertebral body (Figure 4). Histologic morphologic features similar to the primary tumor were found, and because of the penetration of the posterior cortex with intact tumor capsule (but microscopic focal infiltration), the resection was considered marginal as well. The tumor cells did not show any response to preoperative chemotherapy as found in the macroscopy and microscopy pathology. The postoperative course was again completely uneventful.

**Figure 5:**
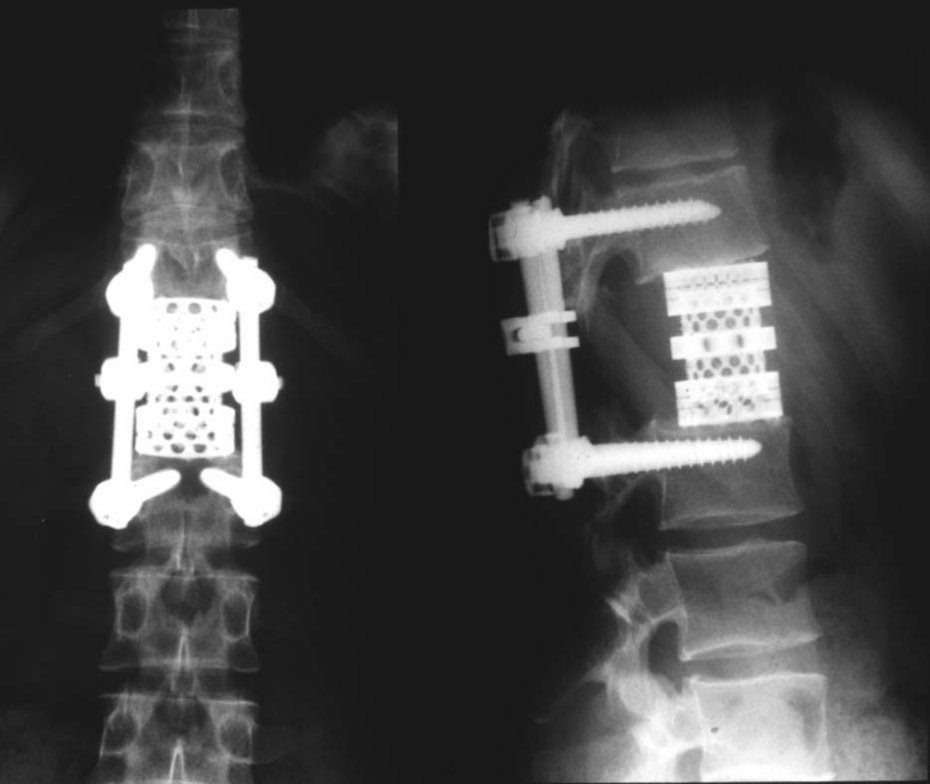
**Anteroposterior and lateral plane radiograph at 10-year follow-up showing no signs of lysis or cage dislocation**.

Because of the marginal resection and the poor response to preoperative chemotherapy, postoperative radiation therapy was added with a dose of 50 Gy. Ten years postoperatively, the now 26-year-old female patient is in excellent general condition without signs of local recurrence or further distant metastasis. Concerning instrumented fusion, no signs of lysis around the pedicle screws or signs of cage dislocation have been detected (Figure 5). CT has revealed that the autologous bone within the cage is mineralized and has most probably fused with the adjacent endplates of the T12 and L2 vertebral bodies, as far as this can be evaluated with this or any other imaging technique.

## Discussion

Paraganglia (or glomus bodies) are extra-adrenal rests of neural crest-derived cells that are closely associated with the autonomic nervous system. They are found in disparate areas of the body, including the head, neck, thorax, abdomen and retroperitoneal space. Paragangliomas arising from carotid bodies appear to have the highest propensity for metastatic spread to the spine [1]. The retroperitoneal extra-adrenal paraganglioma is the most aggressive one with malignant behavior in up to 50% of the cases [4,5]. So far no publications have come to our attention that predict clinical outcome of patients with paraganglioma by conventional histology. Therefore, malignancy can only be demonstrated by the presence of chromaffin tissue at sites where it is usually not present, such as bone, lung or liver, or local recurrence after total resection of a primary mass. In this case, staging after resection of the primary tumor revealed a solitary metastasis in the vertebral body of L1. This is unusual because metastases have been reported to occur usually intradurally when the spine is involved [3,11,13,18,19]. Isolated metastatic involvement of vertebral bodies is extremely rare, and only isolated case reports have been published. Brodkey et al [1] presented the case of a 54-year-old man with a metastatic lesion in the body of C2, which was resected. They did not mention whether the procedure was intralesional or marginal. Over a 30-month period, the patient's myelopathy resolved, and there had been no progression of the disease.

Razakaboay et al [20] reported on three patients who developed bone metastasis of a retroperitoneal paraganglioma occurring up to 17 years after resection of the primary tumor. The treatment of choice was surgery and radiotherapy.

A third case was published by Hamilton and Tait [21], who described metastatic retroperitoneal paraganglioma associated with spinal cord compression in two young men. One was metastatic at presentation, and the other became metastatic 19 years after surgical resection of the primary tumor. Both men died because of widespread metastatic disease.

The latest report was published by Lehmen et al [22], who described the case of metastatic lesion in a cervical vertebra treated by surgery and adjuvant radiation.

In our case, the superficial intraosseous extension of the tumor within the vertebral body, which occupied zones 4 to 9, according to the staging system of Boriani et al [23], (Weinstein Boriani Biagini (WBB) staging system) made en bloc resection possible. However, because of the destruction of the posterior wall of the vertebral body, only a marginal resection could be obtained. The pseudocapsule was examined and considered intact. With posterior bisegmental transpedicular screw instrumentation using a rigid internal fixator and anterior strut grafting using a modular cage filled with autologous morselized rib graft, a primary stable load-sharing situation could be obtained, and the patient was mobilized without additional external support. Ten years after surgery, the instrumented spine seems to be fused and is absolutely stable.

Chemotherapy was applied before en bloc resection of L1, according to the recommendation of our pediatric oncologists, but histologic microscopy examination of the specimen did not show any response of the tumor cells to the preoperative chemotherapy. This finding is in line with a number of disappointing reports on chemotherapy for this type of tumor [21,24,25], and it must be emphasized that preoperative neoadjuvant chemotherapy seems to be of no value in the treatment of patients with metastatic retroperitoneal paragangliomas.

In a 1992 review, Schild et al [13] showed that radiotherapy is beneficial in the treatment of paragangliomas. Later, postoperative radiotherapy was recommended by several authors [1,2,21].

Therefore, we decided to apply radiotherapy with 50 Gy postoperatively. Ten years after surgery, the patient is without signs of local recurrence or distant metastasis, completely asymptomatic and very satisfied with the result of the operation.

## Conclusion

En bloc resection of a solitary metastatic paraganglioma combined with postoperative radiotherapy seems to be the ideal and only curative therapeutic modality, which is in line with other report on the treatment of specific solitary metastasis as well as primary tumors of the spine [2,23,26-30]. Chemotherapy is without any value, according to the literature and our own experience, and therefore should not be recommended. With posterior short segmented transpedicular screw instrumentation and anterior strut grafting using a modular cage filled with morselized autologous bone grafts, primary and long-term stable instrumented fusion can be obtained. Patient outcome in this case with a disease-free interval of now 10 years at present strongly justifies en bloc spondylectomy and instrumented reconstruction in a solitary paraganglioma metastasis of a vertebral body. Because of descriptions of recurrence up to 19 years [20,21] after primary tumor resection, further surveillance screening (including 24-hour urinary fractionated metanephrines and catecholamines) is recommended.

## Competing interests

The authors declare that they have no competing interests.

## Consent

Written informed consent was obtained from the patient for publication of this case report and any accompanying images. A copy of the written consent is available for review by the Editor-in-Chief of this journal.

## Authors' contributions

AR and HFH contributed to this case report's conception and design. They also performed the literature research, prepared the manuscript and reviewed it for publication. URL, TL and MQ were involved in the literature review and helped draft parts of the manuscript. MQ supervised the writing of the manuscript. URL and HFH performed the operation. HFH, URL and TL supervised the general management and follow-up of the patient. All authors have read and approved the final manuscript.
